# The association between Toll-like receptor 2 single-nucleotide polymorphisms and hepatocellular carcinoma susceptibility

**DOI:** 10.1186/1471-2407-12-57

**Published:** 2012-02-07

**Authors:** Xie Junjie, Jiang Songyao, Shi Minmin, Song Yanyan, Shen Baiyong, Deng Xiaxing, Jin Jiabin, Zhan Xi, Chen Hao

**Affiliations:** 1 Department of Surgery, Ruijin Hospital, Shanghai Jiao Tong University School of Medicine, Shanghai, P.R. China; 2 Research Institute of Digestive Surgery, Ruijin Hospital, Shanghai Jiao Tong University School of Medicine, Shanghai, P.R. China; 3 Department of Pharmacology and Biostatistics, Institute of Medical Sciences, Shanghai Jiao Tong University School of Medicine, Shanghai, P.R. China; 4 Department of Surgery and Research Institute of Digestive Surgery, Ruijin Hospital, Shanghai Jiao Tong University School of Medicine, Shanghai, P.R. China

## Abstract

**Background:**

Toll-like receptors (TLR) are key innate immunity receptors participating in an immune response. Growing evidence suggests that mutations of TLR2/TLR9 gene are associated with the progress of cancers. The present study aimed to investigate the temporal relationship of single nucleotide polymorphisms (SNP) of TLR2/TLR9 and the risk of hepatocellular carcinoma (HCC).

**Methods:**

In this single center-based case-control study, SNaPshot method was used to genotype sequence variants of TLR2 and TLR9 in 211 patients with HCC and 232 subjects as controls.

**Results:**

Two synonymous SNPs in the exon of TLR2 were closely associated with risk of HCC. Compared with those carrying wild-type homozygous genotypes (T/T), risk of HCC decreased significantly in individuals carrying the heterozygous genotypes (C/T) of the rs3804099 (adjusted odds ratio (OR), 0.493, 95% CI 0.331 - 0.736, *P *< 0.01) and rs3804100 (adjusted OR, 0.509, 95% CI 0.342 - 0.759, *P *< 0.01). There was no significant association found in two TLR9 SNPs concerning the risk of HCC. The haplotype TT for TLR2 was associated significantly with the decreased risk of HCC (OR 0.524, 95% CI 0.394 - 0.697, *P *= 0.000). Inversely, the risk of HCC increased significantly in patients with the haplotype CC (OR 2.743, 95% CI 1.915 - 3.930, *P *= 0.000).

**Conclusions:**

These results suggested that TLR2 rs3804099 C/T and rs3804100 C/T polymorphisms were closely associated with HCC. In addition, the haplotypes composed of these two TLR2 synonymous SNPs have stronger effects on the susceptibility of HCC.

## Background

Toll-like receptors (TLRs) belong to a family of trans-membrane receptors that play a key role in immune response against microbial pathogens by recognizing specific microbial molecular components. After activated, TLRs initiate a signaling cascade resulting in the stimulation of innate and adaptive immune responses targeting the invading pathogen [1].

There are some evidences showing potential association between the activation of TLR2/TLR9 and carcinogenesis. Lipopolysaccharide induces the production of transforming growth factor-beta and hepatocyte growth factor mediated by CD14/TLR2 in cultured human colon cancer cell lines [2]. Listeria monocytogenes could activate mitogen-activated protein kinases and nuclear factor-kappaB in tumor cells, resulting in the increased production of nitric oxide and interleukin-6 and enhanced proliferation of tumor cells. All of these effects could be abrogated by silencing expression of TLR2 [3].

TLR signals exist in hepatitis B, hepatitis C, alcoholic liver disease, non-alcoholic liver diseases, primary biliary cirrhosis, primary sclerosing cholangitis, hepatic fibrosis, ischemic-reperfusion injury and liver allograft rejection [4]. TLRs can be expressed by various cell types in the liver. Kupffer cells express TLR2 and TLR9. And hepatocytes and biliary epithelium express TLR2 while stellate cells express TLR9 [4]. TLR2 is downregulated in hepatocytes, Kupffer cells and peripheral blood monocytes in hepatitis-B-antigen-positive patients [5]. In hepatitis-B-e-antigen-negative patients, TLR2 expression and tumor necrosis factor-α production is up-regulated and probably mediated by precore proteins [5]. In vitro, HBV replication could be inhibited by stimulating IL-1 receptor and TLR2 receptors and subsequently activation of a signaling cascade in hepatoma cell lines [6]. In a chemical-induced mouse model developed by Oliva et al. [7], it was shown that tumorigenesis in liver was associated with TLR2 activation. The expression of TLR 9 (CpG-specific) was also found in some hepatoma cell lines such as HepG2, HuH7, and Hep3B. CpG DNA of HBV DNA may contribute to the malignant transformation of benign liver cells [8]. Cell surface stimulation of TLR9 promoted cell proliferation and survival in HCC cell lines [9]. In patients with chronic HBV, TLR9 expression on peripheral blood mononuclear cells was related to HCC [10]. There is growing evidence showing the impact of TLR2 and TLR9 genetic polymorphisms on risk of gallbladder cancer [11], cervical cancer [12], non-Hodgkin lymphoma [13] and endometrial cancer [14].

Based on the information above, we hypothesized that single nucleotide polymorphisms of TLR2 and TLR9 may be related to the occurrence of HCC. To test this hypothesis, a systematic genetic analysis was performed in a Chinese population hospital-based HCC case-control study.

## Methods

### Study design

The data for total 20 TLR2 and 9 TLR9 SNPs genotyped in Chinese (CHD) people population from the database of the International HapMap project http://hapmap.ncbi.nlm.nih.gov/index.html.zh. Fifteen TLR2 SNPs and seven TLR9 SNPs were of minor allele frequencies (MAF) < 5% and were eliminated from subsequent analysis. The remaining 5 TLR2 SNPs were evaluated: two synonymous SNPs in the single exon of the gene (rs3804099, rs3804100) and three SNPs in intron (rs11938228, rs769632 and rs1898830). Two TLR9 SNPs were located in intron (rs352139) and exon (rs352140). Haplotype was analyzed by using a limited number of TLRs SNPs.

### Study samples

We analyzed 211 patients with HCC and 232 non malignant subjects in this case-control study in Ruijin Hospital, Shanghai Jiao Tong University School of Medicine. The ethnic backgrounds of subjects were all Chinese. HCC was diagnosed by the elevation of alpha-fetoprotein (> 400 ng/ml) or by pathological examination in combination with the results of examination of iconography including computer tomography or magnetic resonance imaging and proved to be without bearing any other cancers. Clinical classification was according to International Union Against Cancer (UICC) tumor-node-metastasis (TNM) staging system [15]. For the severity of liver disease, Child-Pugh score was evaluated between two groups.

HBV carriers were defined as positive for both hepatitis B surface antigen and immunoglobulin G against hepatitis B core antigen. The treatments for HCC patients were hepatectomy, liver transplantation, chemotherapy and transcatheter arterial chemoembolization. In order to eliminate the confounding effect of HBV infection in research of genetic susceptibility to HCC, controls were randomly selected from the individuals who attended hepatitis examination in the hospital during the period of case collection. The selection criteria for the control subjects included no medical history of any cancer at the time of ascertainment and frequency matching to the patients on age and gender. All subjects provided informed consent in this study. The study protocol was approved by the independent ethics committee of Ruijin Hospital, Shanghai Jiao Tong University Medical School.

### Genotyping

Genomic DNA was extracted from peripheral blood leukocytes. The genomic regions of interest were amplified by multiplex polymerase chain reactions (PCRs). The PCR reactions were performed and all 7 SNPs were genotyped by SNaPshot method according to our previous protocol [16].

Genotype analysis was performed in a blinded manner so that staff was unaware of the cases or control status. For quality control, a 10% masked random sample of cases and controls was tested repetitively by different investigators and all the results were completely concordant.

### Statistical analysis

Data were expressed as proportions, mean and standard deviation (SD) or medians (range). All these tests were performed with the SPSS 13.0 version for Windows. All reported *P *values were two sided. Grouped data were compared by the Mann-Whitney *U *test.

The Hardy-Weinberg equilibrium (HWE) test using *χ*^2 ^analysis was done for each SNP among cases and controls. Genotype frequency differences were tested between HCC patients and control subjects were tested for each SNP by the *χ*^2 ^test with 2 degrees of freedom.

Odds ratios (ORs) of HCC for the variant-allele carriers (homozygous and heterozygous) versus homozygous wild-type allele carriers were estimated by unconditional logistic regression and adjusted for sex and age (< = 55 or > 55).

Haplotype block structure and the estimates of pair-wise linkage disequilibrium (LD) (D') were determined by using Haploview software http://www.broadinstitute.org/scientific-community/science/programs/medical-and-population-genetics/haploview/haploview. The LD patterns from HapMap CHD population were evaluated to avoid SNPs in strong LD (r^2 ^> = 0.8). Haplotype frequency was estimated with the statistical method by implementing the computer program PHASE. A global score test was used to assess the difference in haplotype frequency distributions between cases and controls. Association between the haplotypes and HCC was performed with the *χ*^2 ^test.

False-positive report probabilities (FPRPs) for the significant results (*P *< 0.05) were calculated to account for potential false positives. FPRP is defined as the probability of no true association between genetic variants and disease given the statistically significant finding. The values of FPRP were assessed by using the method described by Wacholder et al. [17]. The FPRP was determined by: the prior probability of a true association, observed *P *value and statistical power. Moderate-high prior probabilities of 0.10 - 0.25 were assigned and a significant finding with a FPRP < 0.50 was considered.

## Results

The clinical characteristics of HCC patients and control subjects were summarized in Table 1 and no significant differences were observed in age, sex, Child-Pugh score and HBV carriers between two groups (*P *> 0.05).

**Table 1:** Main demographic and clinical characteristics of the studied population

Characteristics	HCC n = 211	Controls n = 232	*P *values
Age, year^a^	53.87 (18 - 90)	47.07 (22 - 90)	0.309
Male gender, n (%)	178 (84.36%)	187 (80.6%)	0.300
HBV carriers, n (%)	172 (81.52%)	184 (79.3%)	0.559
Child-Pugh score			0.202
A	185 (87.68%)	212 (91.38%)	
B	26 (12.32%)	20 (8.62%)	
α-FP level			
> 400 ng/ml, n (%)	78 (36.97%)		
< 400 ng/ml, n (%)	133 (63.03%)		
UICC classification			
Stage I-II, n (%)	98 (46.45%)		
Stage III-IV, n (%)	113 (53.55%)		

^a^Median (range)

In both HCC patients and control group, the genotypes of selected polymorphisms of TLR2 and TLR9 were in Hardy-Weinberg equilibrium, with no significant *χ*^2 ^values (*P *> 0.05) except for rs3804099 and rs3804100 in control group (*P *= 0.012). Although rs3804099 and rs3804100 were deviated from Hardy-Weinberg equilibrium, we still retained them in the analyses as these *P *values were marginal and may be chance findings and the internal blinded quality-control specimens did not show evidence of genotyping error.

**Figure 1:**
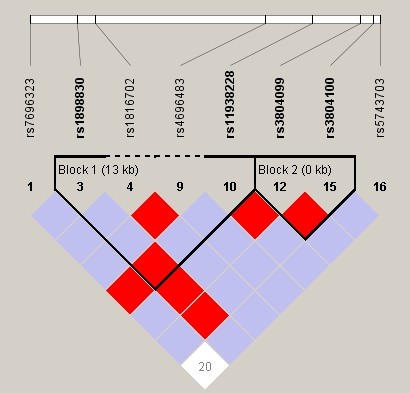
**Linkage disequilibrium plot of TLR2 SNPs in patients with HCC**. D' corresponding to each SNP pair are expressed as a percentage and shown within the respective square. Higher D' is indicated by a brighter red.

The two TLR2 polymorphisms were in high LD (rs1898830 + rs11938228 and rs3804099 + rs3804100, D' > 0.9) (see Figure 1).

The genotype frequencies were compared between the patients and controls for the polymorphisms in TLR2 and TLR9 (see Table 2). Only two TLR2 polymorphisms (rs3804099 and rs3804100) showed significantly different distributions between HCC patients and the controls (*P *< 0.05) (see Table 2). No different distribution between HCC patients and the controls was observed in TLR9 SNPs.

**Table 2:** Genotype distribution of TLR2 and TLR9 SNPs in HCC patients and controls

	SNP	Location	Genotype	HCC	Control	*P *values
**TLR2**	rs7696323	intron	C/C	109	125	0.239
			C/T	80	93	
			T/T	22	14	
	rs1898830	intron	G/G	72	80	0.093
			A/G	92	118	
			A/A	47	34	
	rs11938228	intron	A/A	78	84	0.211
			A/C	89	113	
			C/C	44	35	
	**rs3804099**	**cds-synon**	**C/C**	**121**	**100**	**0.002**
			**C/T**	**71**	**117**	
			**T/T**	**19**	**15**	
	**rs3804100**	**cds-synon**	**C/C**	**130**	**111**	**0.003**
			**C/T**	**67**	**110**	
			**T/T**	**14**	**11**	
**TLR9**	rs352140	cds-synon	C/C	96	102	0.153
			C/T	85	109	
			T/T	30	21	
	rs352139	intron	A/A	82	101	0.362
			G/A	102	110	
			G/G	27	21	

*P *value was calculated by a *χ*^2^-test 3 × 2 contingency table (df = 2)

Carriers of the C/T of rs3804099 and rs3804100 of TLR2 had a nominally significant association with decreased HCC risk (OR 0.493, 95% CI 0.331 - 0.736; OR 0.509, 95% CI 0.342 - 0.759) by age and sex adjustment (*P *< 0.01) (see Table 3) compared with T/T carriers. No significant association was found in TLR9 polymorphisms with the risk of HCC. Furthermore, we evaluated the effect of TLR2 and TLR9 SNPs for α-FP level and UICC pathologic stage in patients with HCC (see Table 4) and found that no SNP had any influence on these two factors.

**Table 3:** Association between hepatocellular carcinoma and TLR2 and TLR9 SNPs

Gene	SNP	Genotype	Odds Ratio (95% CI)	*P *Value	Odds Ratio (95% CI) †	*P *Value
**TLR2**	rs7696323	C/C	1		1	
		C/T	1.014 (0.684 - 1.503)	0.946	1.001 (0.673 - 1.489)	0.995
		T/T	0.555 (0.271 - 1.137)	0.108	0.560 (0.271 - 1.157)	0.118
	rs1898830	A/A	1		1	
		A/G	0.866 (0.570 - 1.317)	0.502	0.902 (0.591 - 1.378)	0.634
		G/G	1.536 (0.891 - 2.647)	0.122	1.531 (0.885 - 2.649)	0.128
	rs11938228	C/C	1		1	
		A/C	0.848 (0.560 - 1.284)	0.437	0.884 (0.582 - 1.344)	0.565
		A/A	1.354 (0.789 - 2.324)	0.272	1.340 (0.777 - 2.309)	0.292
	rs3804099	T/T	1		1	
		**C/T**	**0.502 (0.337 - 0.745)**	**0.001**	**0.493 (0.331 - 0.736)**	**0.001**
		C/C	1.047 (0.506 - 2.166)	0.902	1.048 (0.503 - 2.182)	0.901
	rs3804100	T/T	1		1	
		**C/T**	**0.520 (0.350 - 0.772)**	**0.001**	**0.509 (0.342 - 0.759)**	**0.001**
		C/C	1.087 (0.474 - 2.490)	0.844	0.844 (0.441 - 2.358)	0.964
**TLR9**	rs352140	C/C	1		1	
		C/T	0.829 (0.557 - 1.233)	0.354	0.861 (0.576 - 1.286)	0.464
		T/T	1.518 (0.814 - 2.831)	0.19	1.438 (0.767 - 2.695)	0.258
	rs352139	G/G	1		1	
		G/A	1.142 (0.768 - 1.698)	0.512	1.180 (0.790 - 1.761)	0.419
		A/A	1.584 (0.835 - 3.004)	0.159	1.521 (0.798 - 2.900)	0.202

† Adjusted for age and sex. *P *value was calculated by a *χ*^2^-test 2 × 2 contingency table (df = 1)

**Table 4:** Effect of TLR2 and TLR9 SNPs for α-FP level and UICC pathologic stage in patients with hepatocellular carcinoma

Gene	SNP	Genotype	Odds Ratio (95% CI) †	*P *Value	Odds Ratio (95% CI) ‡	*P *Value
**TLR2**	rs7696323	C/C	1		1	
		C/T	0.623 (0.339 - 1.142)	0.126	0.703 (0.393 - 1.257)	0.235
		T/T	0.639 (0.241 - 1.693)	0.368	0.402 (0.156 - 1.038)	0.060
	rs1898830	A/A	1		1	
		A/G	0.491 (0.771 - 2.885)	0.235	1.434 (0.740 - 2.635)	0.237
		G/G	2.137 (0.994 - 4.597)	0.052	1.304 (0.624 - 2.726)	0.480
						
	rs11938228	C/C	1		1	
		A/C	1.702 (0.894 - 3.239)	0.106	1.421 (0.771 - 2.616)	0.260
		A/A	1.656 (0.764 - 3.587)	0.201	1.458 (0.693 - 3.479)	0.590
	rs3804099	T/T	1		1	
		C/T	1.140 (0.623 - 2.083)	0.671	1.160 (0.644 - 2.088)	0.621
		C/C	0.808 (0.287 - 2.276)	0.686	1.308 (0.492 - 3.479)	0.590
	rs3804100	T/T	1		1	
		C/T	1.192 (0.651 - 2.183)	0.569	1.232 (0.681 - 2.230)	0.490
		C/C	0.706 (0.210 - 2.377)	0.575	0.940 (0.312 - 2.832)	0.913
**TLR9**	rs352140	C/C	1		1	
		C/T	0.928 (0.511 - 1.685)	0.805	0.875 (0.487 - 1.573)	0.655
		T/T	0.465 (0.137 - 1.076)	0.054	0.595 (0.260 - 1.360)	0.218
	rs352139	G/G	1		1	
		G/A	1.106 (0.609 - 2.008)	0.741	1.051 (0.587 - 1.884)	0.866
		A/A	0.470 (0.171 - 1.292)	0.143	0.691 (0.288 - 1.656)	0.407

† α-FP level: > 400 ng/ml or < = 400 ng/ml

‡ UICC classification: stage I, II or stage III, IV

The haplotype analysis was performed to evaluate the frequencies of haplotypes based on the two polymorphisms within the block 2 of TLR2. The haplotype TT was associated significantly with the decrease of the risk of HCC (OR 0.524, 95% CI 0.394 - 0.697, *P *= 0.000). Inversely, the risk of HCC increased significantly in patients with the haplotype CC (OR 2.743, 95% CI 1.915 - 3.930, *P *= 0.000) (see Table 5).

**Table 5:** Association of haplotypes in TLR2 with HCC

Haplotype	Control	Case	OR (95% CI)	*P *Value
	Frequency†	Frequency†		
Block 2				
**TT**	**0.585938**	**0.729748**	**0.524 (0.394 - 0.697)**	**0.000**
TC	0.067079	0.080679	0.779 (0.457 - 1.329)	0.359
CT	0.086476	0.078309	1.099 (0.664 - 1.818)	0.713
**CC**	**0.260507**	**0.111264**	**2.743 (1.915 - 3.930)**	**0.000**

†, proportion of indicated haplotype (%). *P *value was calculated by a *χ*^2^-test 2 × 2 contingency table (df = 1).

The association for two TLR2 polymorphisms and two TLR2 haplotypes showed a FPRP below 0.200, which suggested that these associations are unlikely to represent a false-positive result (see Table 6).

**Table 6:** FPRPs for the selected associations between genetic polymorphisms and HCC

SNP or Haplotype	OR (95% CI)	Observed *P *value	Prior probability			
			
			0.25	0.1	0.01	0.001
SNP						
rs3804099	0.493 (0.331 - 0.736)	0.001	**0.023**	**0.065**	0.434	0.886
rs3804100	0.509 (0.342 - 0.759)	0.001	**0.029**	**0.082**	0.497	0.909
Haplotype (Block 2)						
TT	0.524 (0.394 - 0.697)	0.000	**0.001**	**0.002**	**0.018**	**0.155**
CC	2.743 (1.915 - 3.930)	0.000	**0.001**	**0.002**	**0.018**	**0.155**

## Discussion

The relationship between TLR2 polymorphisms and the progresses of tumors have been explored and reported by some researchers. Boraska Jelavić et al. [18] reported an association of TLR2 GT microsatelite alleles with 20 and 21 GT repeats with sporadic colorectal cancer among Croatians. In the study by Srivastava et al. [19], del allele carriers of TLR2 (Delta22) polymorphism were associated with a 1.54-fold increased risk for gallbladder cancer. In another study by Pandey et al. [12], TLR 2 gene polymorphisms (-196 to -174 del) showed significant association (OR 1.6, 95% CI 1.00-2.51) with cervical cancer susceptibility. Similarly, it was also confirmed in the study by Nischalke et al. [20] that the frequency of the TLR2 -196 to -174del allele was significantly higher in cases with HCV-associated HCC than in HCV-infected patients without HCC. And in carriers of the TLR2 -196 to -174del allele stimulation of monocytes resulted in significantly lower TLR2 expression levels and IL-8 induction than in individuals with the TLR2 -196 to -174ins/ins genotype. The current study was the first one to show that inherited variation in TLR2 influences the risk of HCC. We observed the positive association between two SNPs in exon (rs3804099 and rs3804100) and the risk of HCC. The risk of HCC was found to be significantly low in individuals carrying the heterozygous genotypes of these two SNPs by comparing with those carrying wild-type homozygous genotypes (OR, from 0.331 to 0.759, *P *< 0.001). Our data suggested that TLR2 gene variation may play an important protective role in the occurrence of HCC. Though the observed two-fold decrease in risk is modest, our finding is intriguing because genes in multiple pathways alter the risk for HCC, and each individual gene is likely to contribute only a modest risk.

rs3804099 and rs3804100 of TLR2 are synonymous SNPs. It has long been assumed that synonymous SNPs are inconsequential, as they do not lead to a change in primary polypeptide sequence. However, over the last decade, it has been confirmed in many studies that synonymous mutations are implicated in diseases. Several mechanisms by which synonymous mutations exert their impact on gene function are: (1) Perturbations of mRNA splicing. Ectopic mRNA splicing generated by synonymous SNPs can bring about distinct phenotypes, leading to human disease [21]. Synonymous base pair changes in auxiliary elements within exons (exonic splicing enhancers and exonic splicing silencers) can directly change the splicing patterns of mRNA transcripts, or they can alter the penetrance of concurring mutations elsewhere in the gene [22,23]. (2) The stability of mRNA. Synonymous SNPs can affect the stability of mRNA via cis factors which determine mRNA stability fall within the 3'-untranslated region of a transcript [24]. (3) mRNA structure. Synonymous SNPs can modulate mRNA structure and have downstream effects on protein expression and phenotype [25]. (4) Protein folding. It has been confirmed by some computational and experimental studies [26,27] that synonymous SNPs have an influence on protein folding and ultimately protein function. Which mechanism by these synonymous SNPs impact should be investigated in future research.

In the present study, LD analysis showed that these two TLR2 synonymous SNPs were in high LD and located in one haplotype block. Individuals carrying haplotype TT was significantly associated with the decrease of the risk of HCC (OR 0.524, 95% CI 0.394 - 0.697, *P *= 0.000). Inversely, the risk of HCC significantly increased in patients carrying haplotype CC (OR 2.743, 95% CI 1.915 - 3.930, *P *= 0.000). In a recent study by Diatchenko et al. [28], it was shown that the low pain sensitivity (LPS) haplotype composed of synonymous SNPs produces much higher levels of the cathechol-O-methyltransferase (COMT) enzymatic activity when compared with the average pain sensitivity (APS) or high pain sensitivity (HPS) haplotypes. The variation in COMT expression levels among haplotypes was due to the differences in protein translation efficiency, which marks the final manner in which synonymous SNPs can exert their influence on protein translation and cotranslational protein folding. Hunt et al. [24] suggested that haplotypes composed of synonymous SNPs can have profound effects on gene function, and in some cases their effects can be stronger than those of their non-synonymous counterparts.

Few investigations have been reported about the relationship between TLR9 polymorphisms and the tumor susceptivity. In the study by Ashton et al. [14], haplotype analysis showed that the combination of the variant alleles of the two TLR9 polymorphisms, rs5743836 and rs187084, were protective in endometrial cancer (OR 0.11, 95% CI 0.03-0.44). In a case-control study by Etokebe et al. [29], TLR9 (c.1635A > G) polymorphisms were not likely to be a risk factor in developing breast cancer. Although Hold et al. [30] found TLR9 (TLR9-1237 T/C, rs574383) was associated with the development of premalignant gastric lesions in 2006, but their most recent study showed that this SNP didn't increase the risk of gastric cancer itself [31]. Similarly, we didn't find any relationship between two TLR9 genetic variations and the occurrence of HCC.

Recently, Zhang and colleagues [32] conducted a genome-wide association study by genotyping 440,794 SNPs in 355 chronic HBV carriers with HCC and 360 chronic HBV carriers without HCC in Chinese population using Affymetrix Genome-Wide Human SNP Array 5.0. In this study, one intronic SNP (rs17401966) in KIF1B on chromosome 1p36.22 was highly associated with HBV-related HCC. However, only rs11938228 and rs10759930 of TLR2 and rs1927911 of TLR9 were enrolled in Affymetrix Genome-Wide Human SNP Array 5.0 chip and these three SNPs were not related to HCC. In our study, only TLR2 rs11938228 was determined and our result was consistent to them, that TLR2 rs11938228 was not associated with the risk of HCC.

Although SNP rs3804099 and rs3804100 were out of HWE (*P *= 0.01 - 0.02), we retained them in the analyses as these *P *values were marginal and may be chance findings and the internal blinded quality-control specimens did not show evidence of genotyping error. These two SNPs genotyped were deviated HWE in the controls but not in the cases. The association for these two polymorphisms showed a FPRP below 0.200. It suggested strongly that these associations are unlikely to represent a false-positive result. Considering these two SNPs showed any evidence of deviation in cases, it was not believed that the marginal HWE tests in controls suggest systematic genotyping errors.

## Conclusions

TLR2 rs3804099 C/T and rs3804100 C/T polymorphisms were associated closely with HCC. In addition, the haplotypes composed of these two synonymous SNPs have profound effects on the susceptibility of HCC.

## Competing interests

The authors declare that they have no competing interests.

## Authors' contributions

XJ and JS carried out the genotyping analysis, and drafted the manuscript. SM participated the collection of clinical materials. SY performed the statistical analysis. SB, DX participated in the design of the study. JJ and ZX helped to draft the manuscript. CH designed the study, participated the analysis of the results and drafted the manuscript. All authors read and approved the final manuscript.

## Pre-publication history

The pre-publication history for this paper can be accessed here:

http://www.biomedcentral.com/1471-2407/12/57/prepub
